# Fatal Monocytic Ehrlichiosis in Woman, Mexico, 2013

**DOI:** 10.3201/eid2205.151217

**Published:** 2016-05

**Authors:** Carolina G. Sosa-Gutierrez, Fortino Solorzano-Santos, David H. Walker, Javier Torres, Carlos A. Serrano, Guadalupe Gordillo-Perez

**Affiliations:** Unidad de Investigación de Enfermedades Infecciosas y Parasitarias, Mexico City, Mexico (C.G. Sosa-Gutierrez, F. Solorzano-Santos, J. Torres, G. Gordillo-Perez);; University of Texas Medical Branch, Galveston, Texas, USA (D.H. Walker);; Hospital Infantil de México Federico Gomez, Mexico City (C.A. Serrano)

**Keywords:** *Ehrlichia chaffeensis*, human monocytic ehrlichiosis, bacteria, vector-borne infections, ticks, fatal, Mexico

## Abstract

Human monocytic ehrlichiosis is a febrile illness caused by *Ehrlichia chaffeensis*, an intracellular bacterium transmitted by ticks. In Mexico, a case of *E. chaffeensis* infection in an immunocompetent 31-year-old woman without recognized tick bite was fatal. This diagnosis should be considered for patients with fever, leukopenia, thrombocytopenia, and elevated liver enzyme levels.

*Ehrlichia* are rickettsia-like intracellular bacteria of human medical and veterinary importance. The first cases of human monocytic ehrlichiosis (HME) were described in 1987, and the etiologic agent was subsequently identified in the United States as *Ehrlichia chaffeensis*, a strictly intracellular bacterium belonging to the family *Anaplasmataceae (**1*). *E. chaffeensis* is transmitted by *Amblyomma americanum* ticks; reservoirs include domestic and wild animals (*2*,*3*). In the United States, most cases occur from April through September. Ehrlichiosis is usually self-limiting with nonspecific symptoms similar to those of influenza: fever, malaise, headache, and myalgia. Leukopenia is found in 60%–70% of patients, thrombocytopenia in 60%, and mild to moderate elevation of serum transaminase levels in 80%–90% (*4*,*5*). Serologic evidence of infection may be absent during the early acute phase of illness. In patients with illness severe enough that they seek medical attention, 50% require hospitalization and 2%–3% die (*6*).

In Mexico, only 1 case of *E. chaffeensis* has been reported (*7*). Recently, *E. chaffeensis* has been identified in *Rhipicephalus sanguineus* and *Amblyomma cajenennse* ticks, which are found throughout Mexico (*8*). We report another case in Mexico, this one fatal.

## The Study

In late August 2013, a previously healthy 31-year-old woman from Estado de Mexico, in central Mexico, was admitted to an emergency department with a history of fever for 15 days, chills, muscle aches, malaise, loss of appetite, and headache. She had worked in a marketplace selling fruit and vegetables; she had not traveled abroad in the previous 3 months and was not aware of having been bitten by a tick. At the time of physical examination, she was confused and had mild respiratory distress, hepatosplenomegaly, tachycardia, and blood pressure within reference range. Blood collected at the time of admission showed leukopenia (0.90 × 10^9^ cells/L), neutropenia (0.31 × 10^9^ cells/L), lymphocytopenia (0.59 × 10^9^ cells/L), thrombocytopenia (76 × 10^9^ platelets/L), anemia (hemoglobin 8.2 g/dL), and elevated serum concentrations of aspartate transaminase (2,748 IU/L) and alanine transaminase (350 IU/L). A full evaluation for sepsis (blood cultures, morphologic evaluation, and culture of bone marrow aspirate) was performed. The bone marrow aspirate contained no significant abnormalities. Computed tomography indicated hepatosplenomegaly and a small pericardial effusion; ultrasonography indicated bilateral nephromegaly; and echocardiography indicated a small pericardial effusion and an ejection fraction of 59%. 

**Figure 1:**
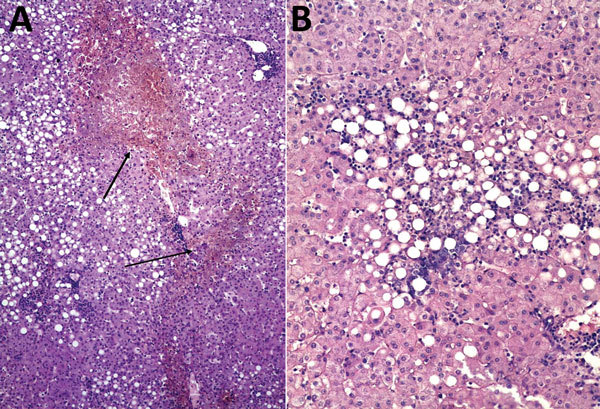
Histopathologic appearance of liver biopsy sample from woman with fatal human monocytic ehrlichiosis, Mexico, 2013. A) Necrotic hepatic lesions in a patchy distribution (arrows). Hematoxylin and eosin (H&E) stain; original magnification ×100. B) Macrovesicular steatosis and inflammatory lymphocytic infiltrate. H&E stain; original magnification ×200.

After these procedures were completed, the patient was transferred to the intensive care unit (ICU); 1 day later, she was stable and discharged to a regular hospital ward, at which time blood and bone marrow culture results were negative. No morulae were detected in smears of peripheral blood and bone marrow. At that time, the patient’s mental status included confusion, a psychotic episode, and symptoms of anxiety; a psychiatrist prescribed benzodiazepines. Prednisone therapy was added for suspected hemophagocytic syndrome. The patient’s condition deteriorated; she experienced bleeding and hemodynamic instability and persistent fever. Four days after initial ICU discharge, she was transferred back to ICU, where she received antimicrobial drug therapy consisting of levofloxacin, amikacin, and meropenem and a transfusion of erythrocytes, plasma, and platelets. On her third day in the ICU, the patient still had pancytopenia and elevated concentrations of aspartate transaminase (674 IU/L) and alanine transaminase (105 IU/L). Blood, liver, and spleen samples were evaluated by PCR for *Mycobacterium* spp., *Rickettsia* spp., *Ehrlichia* spp., and *Anaplasma phagocytophilum*. *E. chaffensis* was found in the blood sample, and a morula-like structure was observed in a liver biopsy sample (Figure 1, panel C). Treatment with doxycycline (100 mg/12 h) was initiated; 2 days later her fever abated, but hypovolemic shock resulting from hemorrhage necessitated mechanical ventilatory assistance. On day 4 after initiation of doxycline, acute renal failure developed and hemodialysis was begun. On day 10, the patient experienced multisystemic failure with hemodynamic instability; despite inotropic support, she died.

Laboratory studies of a blood sample taken on the second day after the patient’s original admission to hospital revealed no antibodies against hepatitis A, B, C, or E; parvovirus B-19; or HIV. PCR for *Mycobacterium* spp. was negative. Histopathologic study of the liver showed centrilobular hepatic necrosis, macrovesicular steatosis, and lymphohistiocytic inflammation (Figure 1).

After death, the diagnosis of HME was confirmed by nested PCR amplification of the 16S rRNA gene from spleen and liver tissues, by use of primers previously described (*8*). Sequencing of the PCR products showed 99.8% homology with *E. chaffeensis* str. Arkansas (GenBank accession no. KT308164). PCR results for *Anaplasma phagocytophilum* and *Rickettsia rickettsii* were negative.

**Figure 2:**
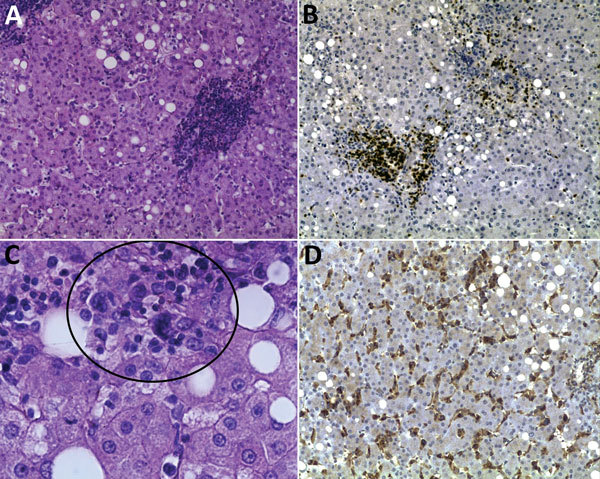
Histopathologic appearance of liver biopsy sample from woman with fatal human monocytic ehrlichiosis, Mexico, 2013. A) Clusters of cells in the liver lobule. Hematoxylin and eosin (H&E) stain; original magnification ×200. B) Immunohistochemical detection of T lymphocytes (CD3). Original magnification ×100. C) Multinucleated cells in parenchyma (circle). H&E stain; original magnification ×400. D) Immunohistochemical detection of macrophages and hyperplasia of Kupffer cells (CD68). Original magnification ×100.

Blood, serum, liver, and spleen samples were transferred to the Rickettsial and Ehrlichial Research Laboratory, University of Texas Medical Branch (Galveston, TX, USA) where 2 fragments of the *dsb* gene were amplified from blood, liver, and spleen DNA by real-time PCR as previously described (*9*). The amplicons showed 100% homology with *E. chaffeensis* str. Arkansas. ELISA and immunofluorescence assays (IFAs) were also performed. Serum antibodies were detected by ELISA (anti–tandem repeat proteins 120 and 32 of *E. chaffeensis*; titer 1:100) and by IFA (IgG; 1:512 titer) (Figure 2).

## Conclusions

Góngora-Biachi et al. previously reported a probable case of HME in Mexico, diagnosed by IFA only (*7*). More recently, *E. chaffeensis* was detected in 5.5% of *Peromyscus* spp. rodents collected from 31 sites in Mexico (*8*). The presence of this pathogen in a wild host is evidence of a tick–vertebrate cycle and represents a potential risk for humans exposed to these tickborne rickettsiae.

The patient we report was hospitalized within 32 days of nonspecific clinical manifestations (leukopenia, anemia, thrombocytopenia, increased serum transaminase concentrations, and hepatosplenomegaly), which have been reported for persistent infection (*10*). Some authors have suggested that the clinical triad of leukopenia, thrombocytopenia, and elevated serum transaminase levels in a febrile patient without a rash is common for patients with HME (*11*). For this patient, multiorgan dysfunction and hematologic abnormalities persisted despite treatment with doxycycline. Death generally results from complications such as acute respiratory distress syndrome or sepsis with multiorgan failure (*12*).

Multiple neurologic manifestations have been reported for patients with ehrlichiosis, including severe headache, confusion, lethargy, hyperreflexia, clonus, photophobia, cranial nerve palsy, seizures, blurred vision, nuchal rigidity, and ataxia (*13*). The patient we report experienced changes in mental status, confusion, and a psychotic episode that was not improved by antimicrobial drug therapy.

To our knowledge, fatal cases of HME have not been reported in Mexico; they may have been ignored by clinicians or they may represent true emergence of the disease in Mexico. In a mouse model, a fatal course of infection has been associated with an *Ehrlichia* strain that induces a toxic shock–like syndrome with high serum levels of tumor necrosis factor α (*14*). Fatal infection is often confounded because the signs and symptoms can mimic findings commonly associated with other infections, such as dengue fever, Rocky Mountain spotted fever, murine typhus, or other misdiagnosed febrile diseases (*15*) that are common in Mexico.

Illness caused by *Ehrlichia* spp. results in nonspecific signs and symptoms, and diagnosis requires a high index of suspicion (*8*). Seasonality should be taken into account; most new cases in humans occur during the summer, when tick activity is highest. Serologic evidence of infection may be absent during the early acute phase of illness (*15*), but use of PCR may help confirm suspected diagnoses. In Mexico, the possibility of *E. chaffeensis* infection should be investigated for patients with febrile illness, leukopenia, thrombocytopenia, and elevated liver enzymes; early diagnosis and timely treatment may prevent death.
